# Expression Profile of Immunoglobulin G Glycosylation in Children With Epilepsy in Han Nationality

**DOI:** 10.3389/fnmol.2022.843897

**Published:** 2022-07-01

**Authors:** Yuejin Li, Fengxue Shi, Guanglei Wang, Jian Lv, Haitao Zhang, Hao Jin, Xueyu Chen, Meng Wang, Peirui Li, Long Ji

**Affiliations:** ^1^Shandong Institute of Parasitic Diseases, Shandong First Medical University & Shandong Academy of Medical Sciences, Jining, China; ^2^School of Clinical, Shandong First Medical University & Shandong Academy of Medical Sciences, Jinan, China; ^3^Tai’an Maternal and Child Health Hospital, Taian, China; ^4^School of Public Health, Shandong First Medical University & Shandong Academy of Medical Sciences, Taian, China; ^5^Department of Critical Care Medical Center, Taian City Central Hospital, Taian, China; ^6^College of Sports Medicine and Rehabilitation, Shandong First Medical University & Shandong Academy of Medical Sciences, Taian, China

**Keywords:** epilepsy, immunoglobulin G, *N*-glycans, children, profile

## Abstract

**Background:**

Epilepsy is a chronic brain disease that recurs during childhood, and more than half of adult epilepsy originates from childhood. Studies suggested that immunoglobulin G (IgG) glycosylation are closely related to neurological diseases. Here we analyzed the characteristics of the immunoglobulin glycosylation profile of children with epilepsy.

**Methods:**

Patients were recruited in Taian, Shandong Province from December 2019 to March 2020. Serum IgG glycome composition was analyzed by hydrophilic interaction liquid chromatography with ultra-high-performance liquid chromatography approach.

**Results:**

The proportion of fucosylated glycans in total IgG glycans was 93.72% in the epilepsy patients, which was significantly lower than that in the control group (94.94%). A lower level of total monogalactosylated and digalactosylated glycans were observed in the epilepsy patients group (30.76 and 40.14%) than that in the controls (36.17 and 42.69%). There was no significant difference between the two groups in bisected GlcNAc glycans and sialylated glycans.

**Conclusion:**

The decrease of core fucosylation and galactosylation may promote the inflammatory reaction of the body and participate in the occurrence of epilepsy in children.

## Background

Epilepsy is a chronic brain disease characterized by repetitive, paroxysmal and transient central nervous system dysfunction caused by excessive discharge of brain neurons (Thijs et al., 2019). It has a high incidence rate, which is next only to cerebrovascular diseases in neurological diseases (Perucca et al., 2018). Epilepsy affects more than 70 million people worldwide, and 60% of patients whose onset of epilepsy occurs in childhood (Vezzani et al., 2016; Trinka et al., 2019). Epidemiological data have revealed the incidence of chinese children is about 5%, and there are about 6 million children with epilepsy (Ding et al., 2021). As a chronic disease, epilepsy requires long-term treatment, which seriously affects the life and study of children, and brings heavy psychological and economic burdens to individuals, families and society (Gao et al., 2015).

Due to the complex pathogenesis of epilepsy, different types of epilepsy have different symptoms and characteristics. Studies have shown that the accumulation of immune inflammatory damage may be the core mechanism of epilepsy (Vezzani et al., 2015; Bazhanova et al., 2021). Immune system dysfunction and inflammatory response are the basic mechanisms that cause excessive excitement of brain nerves (Marchi et al., 2014; Rana and Musto, 2018). In the process of epilepsy caused by different etiologies, inflammatory response may play an important role in all stages, not only as a direct cause, but also as inducing factors and aggravating factors (de Vries et al., 2016).

Immunoglobulin G (IgG), which constitutes 10–20% of plasma proteins, plays an important role in the immune system to recognize and clear foreign antigens and pathogens (Meng et al., 2020). There is a conservative *N*-glycosylation modification site on Asn297 in the constant region of IgG (Hou et al., 2019). *N*-glycosylation is a ubiquitous co- and post-translational modification that enriches protein structure and function (Wang et al., 2019). The state of IgG glycosylation modification is closely related to its physical and chemical properties and biological functions (Li et al., 2019). Changes in blood IgG *N*-glycosylation have been observed in Amyotrophic lateral sclerosis, Multiple sclerosis, Alzheimer’s, and other neurological diseases (Grigorian et al., 2012; Gonçalves et al., 2015; Wuhrer et al., 2015; Costa et al., 2019; Fang et al., 2020; Zhang et al., 2021). Although the mechanisms of IgG glycosylation are well studied, the association of IgG glycosylation with epilepsy is particularly rare. As the abnormal activation of key inflammation is involved in the occurrence of epilepsy, the composition change of IgG glycome regulates the balance of pro-inflammatory and anti-inflammatory. We hypothesized that the abnormal modification of IgG glycome might be one of the influencing factors of epilepsy in children. Therefore, this study aims to profile the IgG *N*-glycome in Chinese epilepsy children and to provide a new idea for the mechanism research and treatment of neurological diseases such as epilepsy.

## Materials and Methods

### Subjects and Sample Collection

From December 2019 to March 2020, we recruited epilepsy patients in Taian, Shandong Province, China, who fulfilled the International League against Epilepsy classification criteria (Fisher et al., 2017) for Children Epilepsy. All case participants must meet the following criteria for inclusion and exclusion: (1) no history of using antiepileptic drugs or immunosuppressants in the past 2 weeks; (2) aged 1 to 14 years; (3) no cardiovascular illness, autoimmune disease, tumor, or infectious disease; and (4) they could all provide blood samples and completed informed consent forms. We enlisted the cooperation of healthy children from the same hospital’s Children’s Nutrition and Health Department.

All healthy children must meet the following inclusion and exclusion criteria: (1) no history of any drugs using in the past 2 weeks; (2) aged 1 to 14 years; (3) no cardiovascular illness, autoimmune disease, tumor, or infectious disease; (4) no symptoms such as fever; and (5) being treated for other mental diseases such as autism were excluded. All study participants provided written informed consent. The study was approved by the Ethics Committee of Tai’an Maternal and Child Health Hospital and was conducted in line with the Declaration of Helsinki.

### Immunoglobulin G *N*-Glycans Analysis

Immunoglobulin G glycan analyses were conducted on participants both in the epilepsy patients and healthy populations. IgG isolation, *N*-glycans release, labeling, and detection were executed as described previously (Liu et al., 2020). Briefly, the samples were transferred to the protein G monolithic plate, for IgG binding and cleaning, followed by PBS washing as previously reported. IgG protein were then eluted with 1 mL of 0.1M formic acid and filtered into the collection plate by a vacuum pump. And 170 μL of 1M ammonium bicarbonate was added to neutralize each sample.

Dried IgG was denatured with 30 μL sodium dodecyl sulfate and 10 μL Igepal-CA630 (4%). The glycans were released with 2 units of PNGase F in 10 μL 5× PBS and incubated at 37°C for 20 h. Then, the incubated samples were evaporated in a vapor dryer (Waters, United States). Right after the completion of this step, released glycans were labeled with 35 μL 2-AB fluorescent labeling mixture, included 2-AB (Sigma, United States), acetic acid (Sigma, United States), sodium cyanoborohydride (NaBH3CN; Sigma, United States), and dimethyl sulfoxide (Sigma, United States) at 65°C for 3 h and then purified, washed, and eluted using hydrophilic interaction liquid chromatography solid phase extraction. Finally, labeled IgG *N*-glycans were analyzed by Hydrophilic interaction high performance liquid chromatography-Ultra performance liquid chromatography (Waters, United States) into 24 glycan peaks (GPs), according to our previous descriptions (Liu et al., 2018). The ACQUITY UPLC Glycan BEH Amide Column (Waters 186004742, United States) was used to analyze *N*-labeled glycans released by 2-AB labeling. The amount of glycans in each peak was expressed as a percentage of total integrated area. The glycan structures of the most abundant glycans per peak were reported previously (Liu et al., 2018). Statistical analyses were performed on each glycan, and also on the summary features of IgG glycome composition, i.e., G0: glycans without galactose, G1: glycans with one galactose, G2: glycans with two galactoses, S: sialic acid, S1: monosialylated, F: fucose, B: bisecting GlcNAc, and Gal-ratio: the relative intensity of IgG fucosylated galactosylation.

### Statistical Analysis

Normal distribution of all analysis results was checked using the Kolmogorov-Smirnov test. Continuous variables underlying the abnormal distribution were represented as the medians (interquartile ranges). The difference of continuous variables between two groups was tested using the Wilcoxon rank-sum test. Statistical analyses were conducted using IBM SPSS statistical software, version 25.0 for Windows (IBM Corp., Armonk, NY, United States). All *p* values reported were two-tailed. A *p* value of less than 0.05 was considered of statistical significance.

## Results

### Characteristics of Study Participants

Clinical characteristics of the study are summarized in Table 1. In total, we recruited 38 epilepsy patients (22 males/16 females, mean age 5.63 years) from Tai’an Maternal and Child Health Hospital, Shandong, China. The 42 controls (25 males/17 females, mean age 6.62 years) were selected according to age and sex. There was no difference in age and sex between the epilepsy group and the control group.

**TABLE 1:** Characteristics of study participants.

Variables	Epilepsy patients	Healthy controls	Statistics	*P*-value
Sample size, *n*	38	42		
**Age, years**				
Mean (SD)	5.63 (2.50)	6.62 (2.23)	*t* = 1.868	0.065
Range (Min-Mix)	9 (3–12)	10 (3–13)		
≥7 years, *n*	12	14	χ^2^ = 0.028	0.867
<7 years, *n*	26	28		
Female, *n* (%)	16 (42.1)	17 (40.5)	χ^2^ = 0.022	0.882
Male, *n* (%)	22 (57.9)	25 (59.5)		

SD, standard deviation; *Statistically significant at significant level of 0.05.

### *N*-Glycan Profiles in Participants

Immunoglobulin G-*N* glycan profiles were analyzed by hydrophilic interaction liquid chromatography with ultra-high-performance liquid chromatography (HILIC-UPLC), where 24 initial GPs (GP1–GP24) were obtained from each chromatogram. The differences of directly measured (GP1–GP24) and derived glycan compositions between epilepsy patients and healthy controls for the Chinese children were shown in Table 2. We found the increased relative abundance of GP1(H3N3F1), GP2(H3N4), GP3(H3N5), GP4(H3N4F1), GP5(H5N2), GP11[H4N5F1(3)], and GP13(H5N5) and the reduced relative abundance of GP7(H4N4), GP8[H4N4F1(6)], GP9[H4N4 F1(3)], GP10[H4N5F1(6)], GP12(H5N4), GP14(H5N4F1), GP15(H5N5F1), GP16[H4N4F1S1(3)], and GP21(H5N4S2) in the epilepsy patients compared with the healthy controls. The initial GPs of GP6(H3N5F1), GP17(H5N4S1), GP18(H5N4F1S1), GP19(H5N5F1S1), GP20(H5N4F2S1), GP22(H5N5S2), GP23(H5N4F1S2), and GP24(H5N5F1S2) were no statistical differences between the two groups.

**TABLE 2:** Serum *N*-glycan levels of epilepsy subjects and control subjects.

*N*-glycan	*N*-glycan structure	Epilepsy subjects (*N* = 38)	Controls (*N* = 42)	*Z*	*P*-value
		
		Median (P25–P75)	Median (P25–P75)		
GP1	H3N3F1	0.16 (0.09–0.47)	0.06 (0.05–0.08)	5.396	<0.001*
GP2	H3N4	0.95 (0.51–1.13)	0.42 (0.33–0.69)	3.632	<0.001*
GP3	H3N5	0.75 (0.35–1.31)	0.26 (0.14–0.43)	3.950	<0.001*
GP4	H3N4F1	23.28 (20.22–26.11)	15.84 (13.34–18.93)	6.397	<0.001*
GP5	H5N2	0.45 (0.17–1.56)	0.13 (0.12–0.15)	5.695	<0.001*
GP6	H3N5F1	3.69 (3.22–4.52)	4.17 (3.52–4.69)	1.513	0.13
GP7	H4N4	0.21 (0.06–0.42)	0.43 (0.30–0.54)	3.739	<0.001*
GP8	H4N4F1(6)	17.42 (13.82–18.75)	19.03 (17.36–19.82)	3.285	0.001*
GP9	H4N4F1(3)	5.23 (3.87–6.26)	9.05 (8.22–9.56)	6.937	<0.001*
GP10	H4N5F1(6)	3.99 (2.59–5.41)	4.74 (4.32–5.17)	2.601	0.009*
GP11	H4N5F1(3)	1.78 (1.50–2.60)	0.51 (0.44–0.59)	7.631	<0.001*
GP12	H5N4	0.68 (0.28–1.17)	1.44 (0.95–1.71)	3.719	<0.001*
GP13	H5N5	0.44 (0.27–0.65)	0.3 (0.27–0.33)	3.160	0.002*
GP14	H5N4F1	17.3 (14.98–19.32)	19.7 (17.31–22.18)	3.478	0.001*
GP15	H5N5F1	1.47 (1.27–2.10)	2.22 (1.76–2.42)	4.239	<0.001*
GP16	H4N4F1S1(3)	2.05 (1.52–2.65)	2.84 (2.42–3.05)	5.010	<0.001*
GP17	H5N4S1	1.07 (0.69–1.68)	1.04 (0.86–1.27)	0.039	0.97
GP18	H5N4F1S1	13.03 (11.57–14.2)	12.87 (10.75–14.05)	0.665	0.506
GP19	H5N5F1S1	1.4 (1.23–1.59)	1.38 (1.25–1.46)	0.973	0.33
GP20	H5N4F2S1	0.41 (0.23–0.59)	0.32 (0.25–0.37)	1.214	0.225
GP21	H5N4S2	0.27 (0.19–0.45)	0.50 (0.45–0.54)	4.577	<0.001*
GP22	H5N5S2	0.06 (0.05–0.24)	0.08 (0.07–0.09)	1.177	0.239
GP23	H5N4F1S2	1.48 (1.17–1.75)	1.63 (1.45–1.78)	1.879	0.06
GP24	H5N5F1S2	1.17 (1.11–1.32)	1.10 (0.94–1.34)	1.754	0.079

GP, glycan peaks; H, Mannose; N, N-acetylglucosamine; F, Fucose; and S, sialic acid. *Statistically significant at significant level of 0.05.

We further calculated the derived glycans traits using the measurement data of the initial GPs, which consist of core fucosylation, bisecting GlcNAc, agalactosylation, monogalactosylation, digalactosylation trait, sialylation traits, and Gal-ratio traits (Qin et al., 2019). As listed in Table 3, 10 of 17 derived glycans were different between epilepsy patients and controls.

**TABLE 3:** Relative abundance (%) of main IgG glycome features in epilepsy patients and the healthy controls.

Summary glycans	Epilepsy subjects	Controls	*Z*	*P*-value
	
	Median (P25–P75)	Median (P25–P75)		
GPN	78.2 (76.4–80.49)	78.71 (76.51–81.08)	0.019	0.985
S1	17.84 (15.91–19.08)	17.8 (15.56–19.65)	0.376	0.707
S2	3.23 (2.76–3.65)	3.23 (3.06–3.66)	0.925	0.355
GPS	21.41 (19.08–23.04)	21.14 (18.72–23.16)	0.231	0.817
G0	28.48 (26.66–32.05)	20.5 (17.62–24.64)	6.359	<0.001*
G1	28.93 (25.17–30.14)	33.46 (32.03–35.25)	6.205	<0.001*
G2	20.4 (18.32–22.93)	24 (20.98–26.25)	3.931	<0.001*
F	93.72 (91.35–95.31)	94.94 (94.08–95.96)	2.833	0.005*
FN	94.56 (93.06–96.39)	96.01 (95.04–97.22)	2.91	0.004*
FS	92.61 (88.3–94.86)	92.41 (91.58–93.21)	0.443	0.658
B	15.33 (14.3–17.41)	14.3 (13.75–16.25)	1.715	0.086
BN	13.74 (12.31–16.58)	12.3 (11.73–14.4)	2.467	0.014*
BS	13.16 (11.84–14.78)	12.66 (10.37–13.69)	1.917	0.055
FG0	23.28 (20.22–26.11)	15.84 (13.34–18.93)	6.397	<0.001*
FG1	22.1 (18.85–24.28)	27.75 (26.51–28.98)	6.484	<0.001*
FG2	17.3 (14.98–19.32)	19.7 (17.31–22.18)	3.478	0.001*
Gal-ratio	0.4 (0.35–0.47)	0.23 (0.18–0.3)	6.744	<0.001*

F, fucose; S, Sialic acid; B, bisecting N-acetylglucosamine (GlcNAc); and G, galactose. *Statistically significant at significant level of 0.05.

### Fucosylation

**FIGURE 1:**
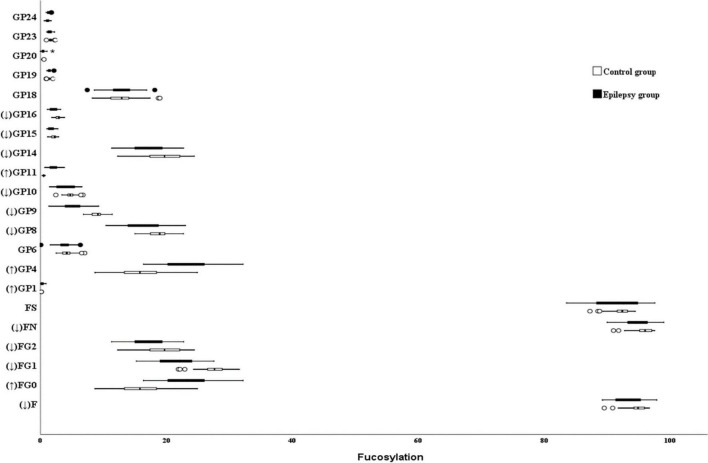
Fucosylation on Ig G in Epilepsy patients and the healthy controls. GP, glycan peak; F, fucose; S, Sialic acid; B, bisecting N-acetylglucosamine (GlcNAc); G, galactose; ↑, the glycan in Epilepsy group in significantly higher than that in the controls; and ↓, the glycan in Epilepsy group in significantly lower than that in the controls. *Represents the extreme values in the sample data.

As shown in Figure 1, 10 fucosylated glycans (GP8, GP9, GP10, GP14, GP15, GP16, FN, FG2, FG1, and F) were significantly lower in the epilepsy patient group when compared to the controls. Meanwhile, 4 fucosylated glycans (GP1, GP4, GP11, and FG0) were significantly higher in the epilepsy patients group when compared to the controls. The proportion of fucosylated glycans in total IgG glycans was 93.72% in the epilepsy patients, which was significantly lower than that in the control group (94.94%; Table 4). Furthermore, we compared the core fucosylation of IgG in different genders and ages. As listed in Supplementary Tables 1, 2, no differences in core fucosylated moieties were identified between the different genders and ages in epilepsy patients.

**TABLE 4:** Relative abundance (%) of main IgG glycome features in ES patients and the healthy controls.

Summary glycans	ES subjects	Controls	*Z*	*P*-value
		
	Median (P25–P75)	Median (P25–P75)		
Core fucosylation	93.72 (91.35–95.31)	94.94 (94.08–95.96)	2.833	0.005*
Bisecting GlcNAc	15.33 (14.3–17.41)	14.3 (13.75–16.25)	1.715	0.086
Agalactosylation	29.04 (27.55–32.47)	20.63 (17.77–24.76)	6.629	<0.001*
Monogalactosylation	30.76 (26.99–32.76)	36.17 (34.75–38.48)	6.523	<0.001*
Digalactosylation	40.14 (37.27–42.59)	42.69 (38.19–46.56)	2.341	0.019*
Sialylation	21.86 (19.55–23.6)	21.47 (19.03–23.49)	0.096	0.923

GlcNAc, N-acetylglucosamine; *Statistically significant at significant level of 0.05.

### Galactosylation

**FIGURE 2:**
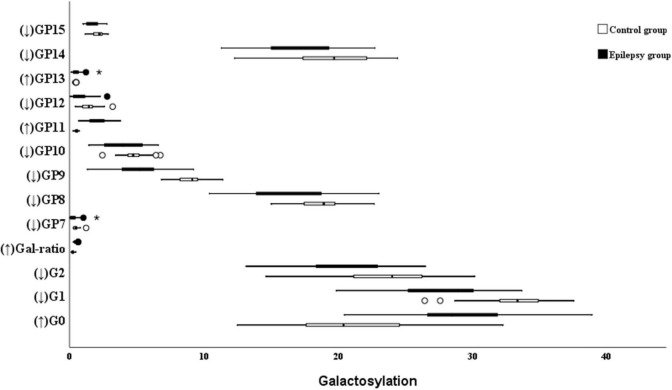
Galactosylation on Ig G in Epilepsy patients and the healthy controls. GP, glycan peak; G, galactose; ↑, the glycan in Epilepsy group in significantly higher than that in the controls; and ↓ the glycan in Epilepsy group in significantly lower than that in the controls. *Represents the extreme values in the sample data.

With regard to galactosylation, the decrease of GP7, GP8, GP9, GP10, GP12, GP14, GP15, G2, and G1 were identified in the epilepsy patients (Figure 2). Therefore, the relative intensity of IgG fucosylated galactosylation (Gal-ratio) and the proportion of agalactosylated glycans in total IgG glycans (G0) were higher in the epilepsy patients when compared to the controls.

The proportion of agalactosylated glycans in total IgG glycans was 29.04% in the epilepsy patients, which was significantly higher than that in the control group (20.63%). Meanwhile, a lower level of total monogalactosylated and digalactosylated glycans were observed in the epilepsy patients group (30.76 and 40.14%) than that in the controls (36.17 and 42.69%; Table 4).

### Sialylation

**FIGURE 3:**
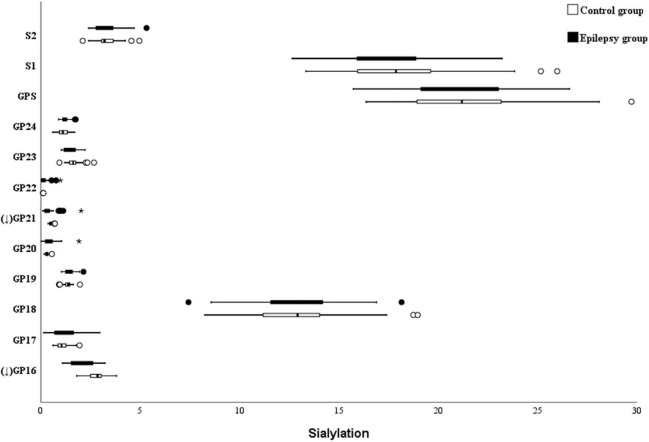
Sialylation on Ig G in Epilepsy patients and the healthy controls. GP, glycan peak; S, Sialic acid; G, galactose; ↑ the glycan in Epilepsy group in significantly higher than that in the controls; and ↓ the glycan in Epilepsy group in significantly lower than that in the controls. *Represents the extreme values in the sample data.

The absence of sialylation IgG reduces its efficacy to complement-dependent cytotoxicity activity *via* C1q binding and leads to an increase in the activation of the lectin-initiated alternative complement pathway (Hou et al., 2021). As shown in Figure 3, the level of sialylated glycans (GP16 and GP21) was significantly lower in the epilepsy patients when compared to the controls. Nevertheless, no difference in the total sialylated glycans was found between the epilepsy patients and controls (21.86 vs. 21.47%; Table 4). Meanwhile, no differences were observed between the different genders and ages in epilepsy patients (Supplementary Tables 1, 2).

### Bisecting GlcNAc

**FIGURE 4:**
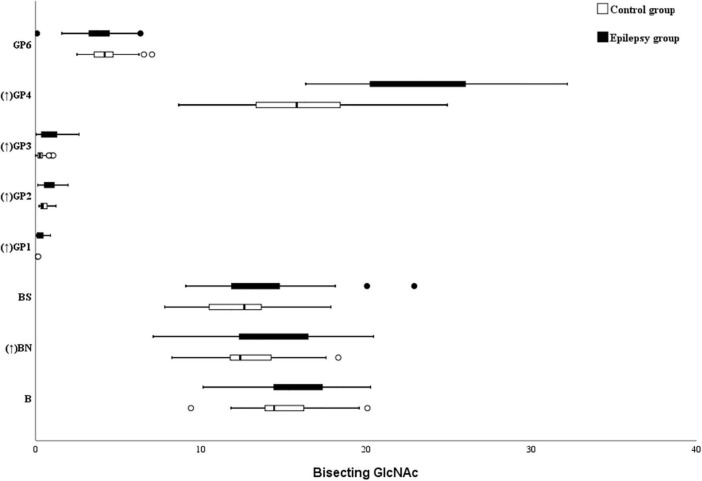
Bisecting GlcNAc on Ig G in Epilepsy patients and the healthy controls. GP, glycan peak; S, Sialic acid; B, bisecting N-acetylglucosamine (GlcNAc); G, galactose; ↑, the glycan in Epilepsy group in significantly higher than that in the controls; and ↓ the glycan in Epilepsy group in significantly lower than that in the controls.

In this study, five bisecting GlcNAc glycans (GP1, GP2, GP3, GP4, and BN) were higher in the epilepsy patients (Figure 4). Nonetheless, as listed in the Table 4, no differences in bisected GlcNAc glycans were identified between the epilepsy patients (15.33%) and controls (14.3%). Further analysis showed that a lower level of total bisected GlcNAc glycans was observed in the female epilepsy children (14.07%) than male (16.15%), however, no differences were identified between the different ages (Supplementary Tables 1, 2).

## Discussion

Whereas studies on glycosylation of human serum IgG as well as nervous system diseases such as Alzheimer’s disease are abundant, IgG glycosylation of epilepsy has been less studied. Particularly in epilepsy, to our knowledge, this is the first study that presents the *N*-glycan profile and investigated the disparate points of the IgG glycome in identifying individuals with epilepsy children using a case–control epidemiological approach.

This study aimed to identify changes in the peripheral serum IgG glycome of children with epilepsy that could represent an interphenotype of the disorder: a link between the genome, the environment, and the disease phenotype. Indeed, this study found that children with epilepsy had a different IgG glycosylation profile than controls.

Observing our classification model, individuals with epilepsy had a reduced relative abundance of GP7, GP8, GP9, GP10, GP12, GP14, GP15, GP16, and GP21, and an increased relative abundance of GP1, GP2, GP3, GP4, GP5, GP11, and GP13. Specifically, the model demonstrated that the presence of epilepsy pathology was predominantly explained with a reduced relative abundance of a galactose *N*-glycan structure and a core fucosylated *N*-glycan structure, as well as an increased relative abundance of a core fucosylated *N*-glycan structure, a bisected GlcNAc *N*-glycan structure and a high-mannitose *N*-glycan structure (GP5).

Epilepsy, as the third common chronic brain disease, is characterized by long-term susceptibility to seizures (Manford, 2017). Numerous evidences showed that immune system dysfunction and inflammatory signals may play a role in various stages of epilepsy development (Matin et al., 2015; Cordero-Arreola et al., 2017; Husari and Dubey, 2019). Pro-inflammatory molecules can change the excitability of neurons and affect the physiological function of glia through paracrine or autocrine, thus disrupting the communication of neurons (Saghazadeh et al., 2014). Interleukin-6 (IL-6) not only regulates immune function, but also affects neuron function, and promotes glial formation and neurogenesis (Erta et al., 2012). For example, Kalueff et al. (2004) found in the mouse model that the increase of IL-6 level in the mouse brain can promote convulsion;Virta et al. (2002) showed that the levels of IL-6 in human plasma and cerebrospinal fluid are significantly increased within 24 h after general compulsive spasm and febrile convulsion.

The Fc segment of Ig G is modified by glycosylation to mediate complement activation, antibody-dependent cell-mediated cytotoxicity (ADCC), etc., thereby regulating inflammation, affecting the body’s pro-inflammatory or anti-inflammatory effects, and then affecting diseases (Russell et al., 2018; Štambuk et al., 2020). The core fucosylation of IgG has been the focus of research because of its role in ADCC (Pucic et al., 2010; Adua et al., 2021). In our latest study, it was shown that the level of IgG fucosylation in COVID-19 patients decreased, which up-regulated ADCC in acute immune response (Hou et al., 2021). In this current study, the IgG core fucosylation level of epileptic children is lower than that of healthy children of the gender-age matched, which indicates that abnormal modification of core fucosylation may be an important biomarker of epilepsy.

The deletion or reduction of galactosylated glycan at the end of IgG makes Ig G exposed at the end of sugar chain to acetylglucosamine, which is not easy to be cleared by phagocytes, leading to the decrease of binding force between antibody and FcγRIIB, activating complement and promoting the occurrence and development of inflammation (Adua et al., 2021). We found that the level of agalactosylation in children with epilepsy is higher, and the levels of monogalactose and digalactose are lower compared to normal healthy children (age-gender matched), which just proves above point. This is not consistent with the results found in the cerebrospinal fluid of patients with amyotrophic lateral sclerosis (Costa et al., 2019).

Sialylation of IgG can inhibit the combination of C1q and galactosylated glycans, thus limiting the pro-inflammatory effect (Zhang et al., 2021). Numerous clinical evidences showed that intravenous immunoglobulin had remarkable curative effect on epilepsy, and the anti-inflammatory activity of sialylation had been found (Dubey et al., 2020; González-Castillo et al., 2020). Although this study discovered that individual sialylation levels in children with epilepsy were lower than those in the control group, there was no significant difference in total sialylation levels, which could be attributed to differences in individual disease states, genetic predispositions, or temporary physiological conditions. Epilepsy has long been considered to be related to some autoimmune diseases (Matin et al., 2015; Cordero-Arreola et al., 2017), and our previous studies found that abnormal modification of IgG *N*-glycans was related to autoimmune diseases such as systemic lupus erythematosus and rheumatoid arthritis (Li et al., 2021).

Higher levels of bisecting GlcNAc on IgG enhance ADCC *via* increased FcγRIII binding and elevate the proinflammatory function of IgG (Hou et al., 2021). Although there is no significant difference in the total bisected GlcNAc glycans between the two groups. Further analysis showed that the bisected *N*-GlcNAc glycans without core fucosed glycan were higher than that of the control group, which indicated that core fucosed glycans and bisected acetylglucosamine have an antagonistic effect. The lack of core fucosed glycans may lead to the weakening of the binding ability of the Fc segment and FcγRIII, enhancing the ADCC effect and triggering pro-inflammatory effects (Hou et al., 2021). At present, however, it is still uncertain whether the enhancement of ADCC is directly caused by the change of bisected *N*-GlcNAc glycans level or caused by the change of core fucosed glycan level (Gao et al., 2017).

These findings provide new insights into the pathogenesis of childhood epilepsy. However, some limitations should be considered: First, as this was a cross-sectional study, our results cannot fully address the causal relationship between IgG glycome and childhood epilepsy. Second, the moderate sample size limits the generalization of conclusions. Third, some relapsed patients may affect the concentration of IgG glycosylation due to the side effects of long-term use of antiepileptic drugs. Despite these limitations, we analyzed the IgG *N*-glycans group in Chinese children with epilepsy, which helps to understand the immune response to childhood epilepsy. This finding, of course, should be confirmed on larger sample size and different stages of epilepsy.

In conclusion, we presented the *N*-glycosylation profile of IgG from epilepsy patients’ plasma and demonstrated that levels of core fucosylation and galactosylation were lower in epilepsy patients than in controls. Meanwhile, the Gal-ratio (the proportion of nogalactosylated glycan in the total) was higher than in healthy children; lack of nogalactosylation can activate the complement system and promote the occurrence of inflammatory response, reduce IgG anti-inflammatory response, and contribute to the pathogenesis of epilepsy. In summary, this study provides new insights into the pathogenesis of epilepsy in children, although further studies are needed.

## Data Availability Statement

The datasets presented in this study can be found in online repositories. The names of the repository/repositories and accession number(s) can be found in the article/Supplementary Material.

## Ethics Statement

The studies involving human participants were reviewed and approved by the Ethics Committee of the Shandong First Medical University & Shandong Academy of Medical Sciences, Taian, China. The ethics approval was given in compliance with the Declaration of Helsinki. Written informed consent to participate in this study was provided by the participants’ legal guardian/next of kin.

## Author Contributions

JL and GW contributed to the study concept and design. FS, HJ, MW, PL, and LJ contributed to the acquisition of subjects and/or data. YL, XC, and HZ contributed to the analysis and interpretation of data. JL, YL, LJ, and XC contributed to the preparation of the manuscript. All authors read and approved the final manuscript.

## Conflict of Interest

The authors declare that the research was conducted in the absence of any commercial or financial relationships that could be construed as a potential conflict of interest.

## Publisher’s Note

All claims expressed in this article are solely those of the authors and do not necessarily represent those of their affiliated organizations, or those of the publisher, the editors and the reviewers. Any product that may be evaluated in this article, or claim that may be made by its manufacturer, is not guaranteed or endorsed by the publisher.

## Abbreviations

IgG: immunoglobulin G
HILIC-UPLC: hydrophilic interaction liquid chromatography with ultra-high-performance liquid chromatography
ILAE: International League against Epilepsy
Fc: fragment crystallizable
ADCC: antibody-dependent cell cytotoxicity
CDC: complement-dependent cytotoxicity
GlcNAc: *N*-acetylglucosamine
GPs: glycan peaks
SD: standard deviation
Gal-ratio: levels of nogalactosylation structure in the total
IL-6: Interleukin-6
F: Fucose
G: Galactose
N: *N*-acetylglucosamine
S: Sialic acid.
