# Supplementary material for: Expression Profile of Immunoglobulin G Glycosylation in Children With Epilepsy in Han Nationality
Source: Front Mol Neurosci. 2022 Jul 1;15:843897. doi: 10.3389/fnmol.2022.843897 (PMC9283856; doi:10.3389/fnmol.2022.843897)
Supplement: Supplementary file 1 [file Data_Sheet_1.docx]

| Supplementary Table S1 Serum *N*-glycan levels of main IgG glycome features in female and male epilepsy patient | | | | |
| --- | --- | --- | --- | --- |
| *N*-glycan | Female (N=44) | Male (N=36) | Z | *P*-value |
|  | Median(P25-P75) | Median(P25-P75) |  |  |
| Core fucosylation | 94.84 (93.66-95.80) | 94.25 (92.50-95.36) | 0.88 | 0.381 |
| Bisecting GlcNAc | 14.07 (13.50-15.98) | 16.15 (14.58-17.47) | 3.30 | 0.001* |
| Agalactosylation | 24.55 (17.90-27.39) | 28.37 (22.58-30.99) | 3.07 | 0.002* |
| Monogalactosylation | 34.29 (30.81-36.01) | 33.66 (30.11-37.45) | 0.11 | 0.911 |
| Digalactosylation | 43.73 (38.83-46.64) | 39.94 (37.19-42.12) | 3.48 | ＜0.001* |
| Sialylation | 22.34 (19.19-23.92) | 20.97 (19.54-22.54) | 1.47 | 0.142 |
| Gal-ratio | 0.29 (0.18-0.34) | 0.38 (0.27-0.44) | 3.15 | 0.002* |

Note: GlcNAc, N-acetylglucosamine; * Statistically significant at significant level of 0.05

| Supplementary Table S2 Serum N-glycan levels of main IgG glycome features in different years group of epilepsy patient | | | | |
| --- | --- | --- | --- | --- |
| *N*-glycan | ≥7 years | ＜7 years | Z | *P*-value |
|  | Median(P25-P75) | Median(P25-P75) |  |  |
| Core fucosylation | 94.81 (93.67-95.46) | 93.76 (91.72-95.48) | 1.264 | 0.206 |
| Bisecting GlcNAc | 14.57 (13.66-16.35) | 15.07 (14.07-16.82) | 1.081 | 0.280 |
| Agalactosylation | 22.83 (19.24-27.61) | 28.64 (25.57-31.08) | 3.32 | ＜0.001* |
| Monogalactosylation | 35.64 (33.13-36.59) | 31.29 (28.01-34.19) | 3.44 | ＜0.001* |
| Digalactosylation | 42.28 (37.68-46.22) | 41.10 (37.57-42.97) | 0.73 | 0.463 |
| Sialylation | 21.44 (19.35-23.47) | 21.86 (18.95-23.56) | 0.23 | 0.817 |
| Gal-ratio | 0.27 (0.20-0.38) | 0.35 (0.29-0.44) | 2.93 | 0.003* |

Note: GlcNAc, N-acetylglucosamine; * Statistically significant at significant level of 0.05
